# Supplementary figures and images for: Fragmented QRS complex in patients with systemic lupus erythematosus at the time of diagnosis and its relationship with disease activity
Source: PLoS One. 2020 Jan 2;15(1):e0227022. doi: 10.1371/journal.pone.0227022 (PMC6939939; doi:10.1371/journal.pone.0227022)

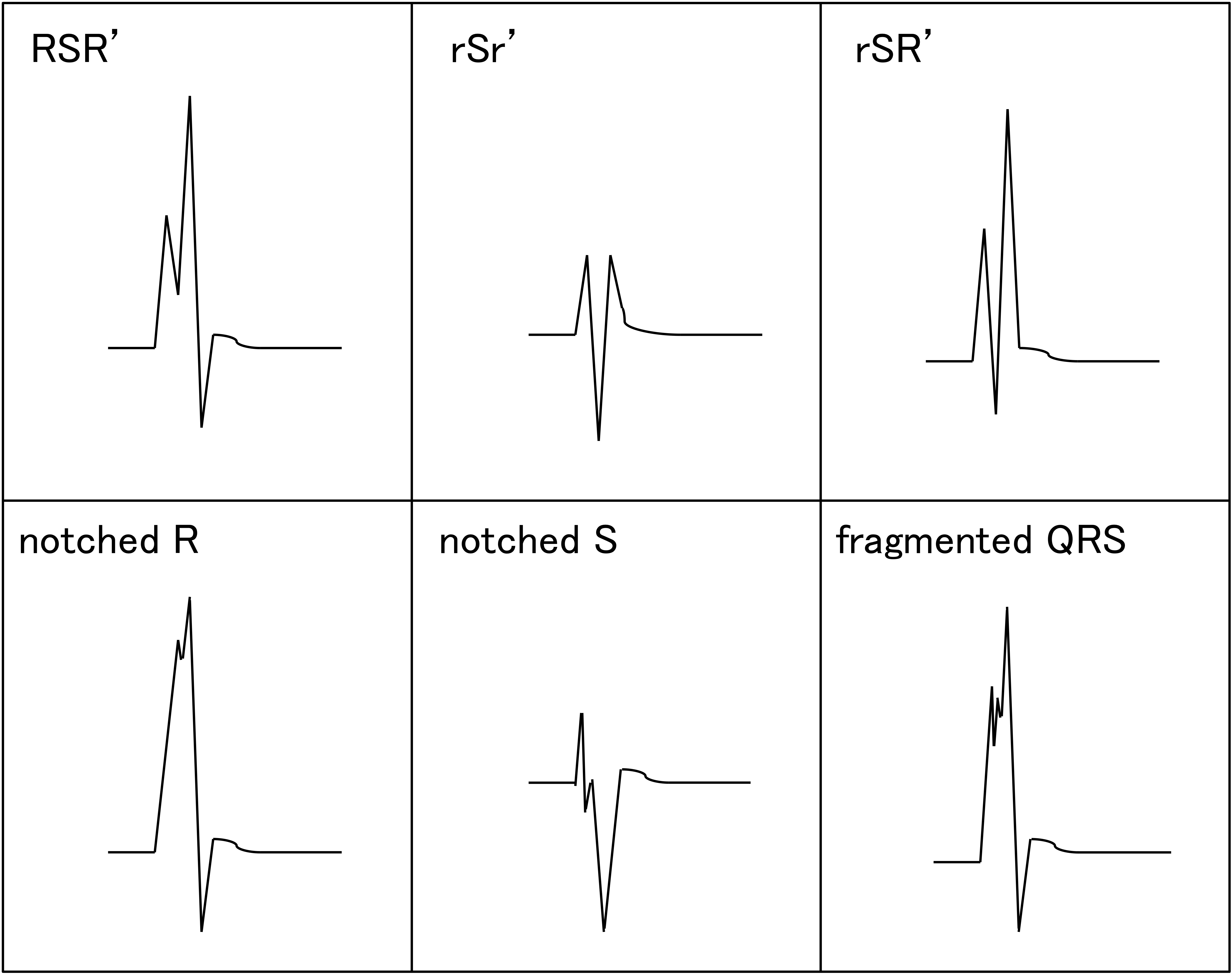

Supplement: S1 Fig — (TIFF) [file pone.0227022.s001.tiff]
