# Supplementary material for: Fragmented QRS complex in patients with systemic lupus erythematosus at the time of diagnosis and its relationship with disease activity
Source: PLoS One. 2020 Jan 2;15(1):e0227022. doi: 10.1371/journal.pone.0227022 (PMC6939939; doi:10.1371/journal.pone.0227022)
Supplement: Attachment — Submitted filename: Response to Reviewers-191206.docx [file pone.0227022.s002.docx]

**Responses to editors’ and reviewers’ comments on PONE-D-19-29766**

We are grateful to Editorial Board Member and two reviewers for their comments and invaluable suggestions. We have incorporated new data after performing additional analysis, and carefully revised the manuscript accordingly. We believe that the revised manuscript, with the changes and new data added, has taken into consideration essentially all of the comments, and hope that will have been improved to the satisfaction of the editors and reviewers. Please find below our point-by-point response to each of the comments made by the reviewers.

**Reviewer comments:**

**Reviewer #1: Hosomuma M et al evaluated relationship between disease activity in SLE patients and fQRS at their diagnosis. Overall the manuscript is on important topic and is written and analyzed with clarity and conviction. However, I have some concerns which authors need to answer.**

**Major**

1. **Authors concluded that a significant association between fQRS presence and disease activity by Table 1 (page 26). Although I agree with author's conclusion by 2.69 of regression coefficient and the low p-value, 95% CI was started with less than 1.0. I wonder their conclusion is always confident.**

We appreciate the opportunity to clarify this point. In case of a multiple linear regression analysis, if the 95% CI contains zero, and the effect will not be significant. On the other hand, In case of a Logistic regression analysis, if the 95% CI contains 1.0, and the effect will not be significant (BMJ 2013;347:f4373). During the main analysis, a multiple linear regression analysis was conducted to assess the association between fQRS and SLE activity, and the 95% CI was not contains zero (0.76–4.61). Therefore, we concluded that it was statistically significant.

2. **As authors mentioned in Introduction section, CVD leads to poor prognosis and early recognition by non-invasive method is highly valuable in clinical settings. By this point of view, this manuscript gives important message. However, my concern is fQRS is really associated with cardiac ischemia or inflammation, systemic disease activity, and future development of CVD. Please show some additional data if authors have conducted further analysis by using Cardiac MRI for patients with or without fQRS, serial change of fQRS after immunosuppressive treatment (Dose it disappear?), and cumulative rate of CVD depending on fQRS presence.**

We appreciate your suggestions. Based on the reviewer’s helpful suggestions, in order to clarify whether it was serial change of fQRS after immunosuppressive treatment, we investigated whether ECG was performed after treatment. Of the 26 patients who were fQRS positive at the time of diagnosis, 18 patients were followed, and 6 patients (33%) disappeared fQRS. This suggests that fQRS was caused by not only irreversible fibrosis but also inflammation and responded to immunosuppressive therapy. Therefore, we think FQRS is associated with acute disease activity. We have included these additional methods, results and related discussion in the revised version of the manuscript (page 7, lines16-17; page 9, lines 14-16; page 11, lines 3-5; page 11, lines 22-page 12, line 3; page 13, lines 15-20).

In the future study, it is necessary to increase the number of cases and clarify what causes the difference between cases where fQRS disappears and cases where it does not. In addition, it has been reported that myocardial inflammation detected by cardiac MRI disappears after treatment (Hinojar R et al. Int J Cardiol. 2016 Nov 1;222:717-726). Unfortunately, we did not measure CMR at the time of diagnosis. In the Limitations section, we have acknowledged this issue.

**Reviewer #2:**

**There are studies on this subject. Therefore, the study does not reveal any new information.**

We appreciate the opportunity to clarify this point.

The novelties of our methods are

#1 using a multivariate analysis adjusted for appropriate confounding factors.

#2 the subject is an SLE at the time of diagnosis that is not affected by treatment.

#3 evaluating the relationship between disease activity and fQRS.

#4 longitudinal assessment of changes in fQRS after treatment.

The novelties of our results are

#1 the first report on an association between ECG abnormalities and disease

　　activity in patients with SLE.

#2 the incidence of fQRS (+) patients who disappeared after immunosuppressive

　　therapy.

**SLE is a chronic disease and FQRS is associated with interstitial fibrosis. Therefore, I do not think FQRS is associated with disease activation.**

Please see our responses to Reviewer #1 Comments 2 described above. Because there are cases where fQRS disappears after treatment, fQRS was caused by not only irreversible fibrosis but also inflammation. Therefore, we think FQRS is associated with acute disease activity.

**Journal Requirements:**

**2. In the ethics statement in the manuscript and in the online submission form, please provide additional information about the patient records used in your retrospective study, including: a) the date range (month and year) during which patients' medical records were accessed; b) the date range (month and year) during which patients whose medical records were selected for this study sought treatment; and c) the source of the medical records analyzed in this work (e.g. hospital, institution or medical center name).**

As requested, we have added this information to the Patient selection section (page 5, lines15-18).

**3. Your ethics statement must appear in the Methods section of your manuscript. If your ethics statement is written in any section besides the Methods, please move it to the Methods section and delete it from any other section. Please also ensure that your ethics statement is included in your manuscript, as the ethics section of your online submission will not be published alongside your manuscript.**

As requested, we have added this information to the Patient selection section (page 5, lines 23-page 6, line 2).

**4. Thank you for stating the following in the Competing Interests section:**

**I have read the journal's policy and the authors of this manuscript have the following competing interests: [Hiroshi Morita is affiliated with the endowment department of Japan Medtronic, Inc. The other authors declare that there is no conflict of interest.]**

**Please confirm that this does not alter your adherence to all PLOS ONE policies on sharing data and materials, by including the following statement: "This does not alter our adherence to PLOS ONE policies on sharing data and materials.” (as detailed online in our guide for authors http://journals.plos.org/plosone/s/competing-interests). If there are restrictions on sharing of data and/or materials, please state these. Please note that we cannot proceed with consideration of your article until this information has been declared.**

As requested, we have added this information to the Declaration of conflicting interests (page 16, lines13-14).
